# Synthesis, Biotransformation, Characterization, and DFT Study of Organic Azachalcone Dyes and Secondary Metabolites with Biological and Conformation Dependence of Dipolar-Octupolar NLO Responses

**DOI:** 10.1021/acsomega.4c09074

**Published:** 2025-03-10

**Authors:** Victoria L. Ribeiro, Neidy S. S. dos Santos, Raira V. S. de Oliveira, Joselina A. Carvalho, Viviane V. Garcia, Hartmann J. S. Brito-Junior, Willibrodus Usfinit, Mayra Pinheiro, Taicia Fill, Rodrigo Gester, Patricio F. Provasi, Sylvio Canuto, Heriberto R. Bitencourt, Patricia S. B. Marinho, Andrey M. R. Marinho

**Affiliations:** † Programa de Pós-Graduação em Química, Universidade Federal do Pará, Rua Augusto Corrêa, 01 - Guamá, 66075-110 Belém, PA, Brazil; ‡ Programa de Pós-Graduação em Química, Universidade Federal do Sul e Sudeste do Pará, 68507-590 Marabá, PA, Brazil; § Instituto de Química, Universidade de Campinas, 13083-970 Campinas, SP, Brazil; ∥ Programa de Pós-Graduação em Ciências Farmacêuticas, Universidade Federal do Pará, Rua Augusto Corrêa, 01 - Guamá, 66075-110 Belém, PA, Brazil; ⊥ Faculdade de Física, Universidade Federal do Sul e Sudeste do Pará, 68507-590 Marabá, PA, Brazil; # Instituto de Física, Universidade de São Paulo, Rua do Matão 1371, 05508-090 São Paulo, SP, Brazil; ∇ Department of Physics, IMIT, Northeastern University, CONICET, AV. Libertad 5500, W 3404 AAS Corrientes, Argentina

## Abstract

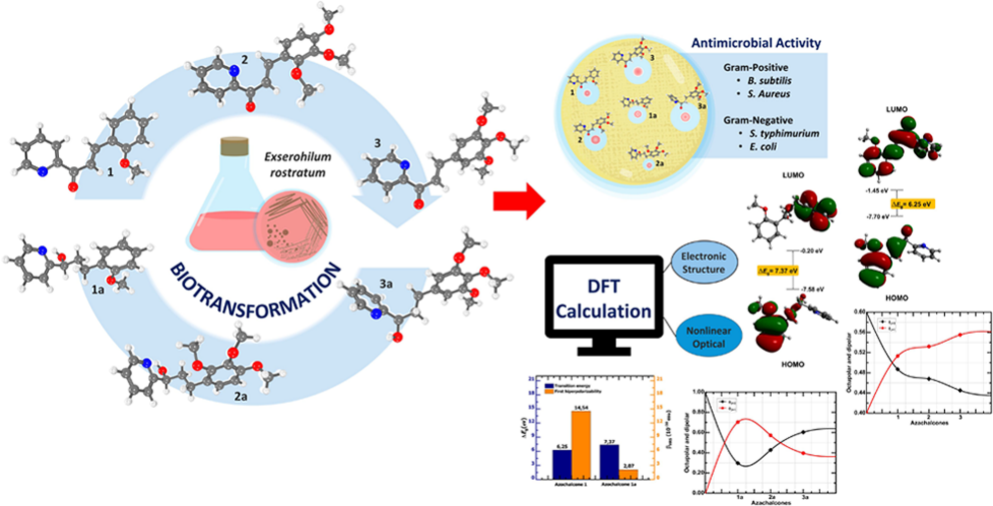

Chalcones are organic chromophores with diverse biological applications and potential for use in various electronic devices due to their recognized optical properties. This research focuses on the organic synthesis, FT-NMR characterization, and biotransformation of three azachalcones (**1**–**3**) using the *Exserohilum rostratum* fungus, yielding novel compounds (**1a**–**3a**). In vitro biological assays against Gram-positive and Gram-negative bacteria revealed promising pharmacological potential for these new chromophores. A key structural difference, the interchange of an HC = CH bond by a H_2_C–CH_2_ bond, significantly impacts biological and electronic properties. For instance, while biotransformed **1a** exhibits similar activity to tetracycline and amoxicillin, compounds **2a** and **3a** demonstrate a 4-fold and thirty-fold increase in inhibitory activity against Gram-negative *E. coli*, respectively, compared to their parent compounds. Density functional theory calculations suggest that the biotransformation reaction reduces the refractive index (*n*), which may limit its applicability in certain light-handling applications. However, Hyper-Rayleigh scattering calculations indicate that these chromophores exhibit higher nonlinear optical (NLO) responses compared to standard NLO materials such as urea and p-nitroaniline, making them promising candidates for photonic and optoelectronic devices, such as nanostructured circuits. Interestingly, while the original molecules exhibit a dominant dipolar (Φ_*J*=1_) NLO response, the biotransformed compounds, as stable isomers, display a predominant octupolar (Φ_*J*=3_) architecture. These findings highlight the potential of these novel compounds for biotechnological and optoelectronic applications.

## 1 Introduction

Chalcones are natural compounds with recognized pharmacological activities, including cytotoxicity and antibacterial activities.^1,2^ Although these dye molecules demonstrate potential biological activity, their primary application lies in nonlinear optics, where they are employed in developing optoelectronic devices such as light-emitting diodes, field-effect transistors, solar cells, and sensors.^3,4^ Due to these functionalities, several chalcones have been synthesized in either case.

Concerning modern pharmacology, since the ’90s, almost 40% of all medicines are developed or inspired on natural product chemical structures from plant or animal sources, often taking advantage of bioreduction reactions, which occur within enzymes extracted from microorganisms.^5,6^

Moreover, frequently producing compounds with enhanced reactive properties, these reactions have the advantage of being green procedures once they do not use solvents that usually damage the environment.^7,8^ Due to these advantages, submitting dye to some enzymatic reaction to obtain new potential pharmaceuticals has become common practice.

**Figure 1:**
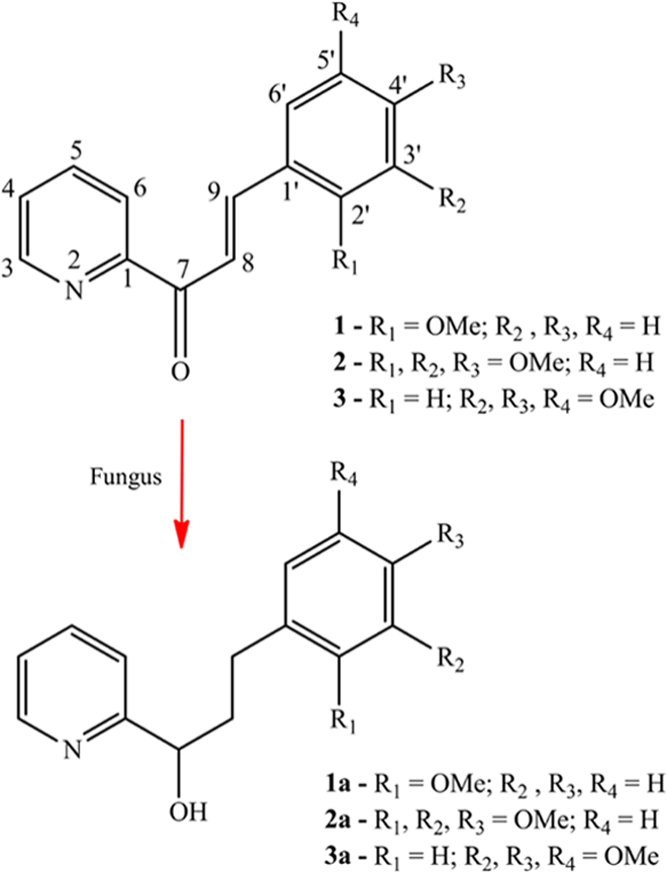
Compounds **1**, **2**, and **3** azachalcones synthesized (top), and their respective bioreduction products **1a**, **2a**, and **3a** obtained (bottom).

**Figure 2:**
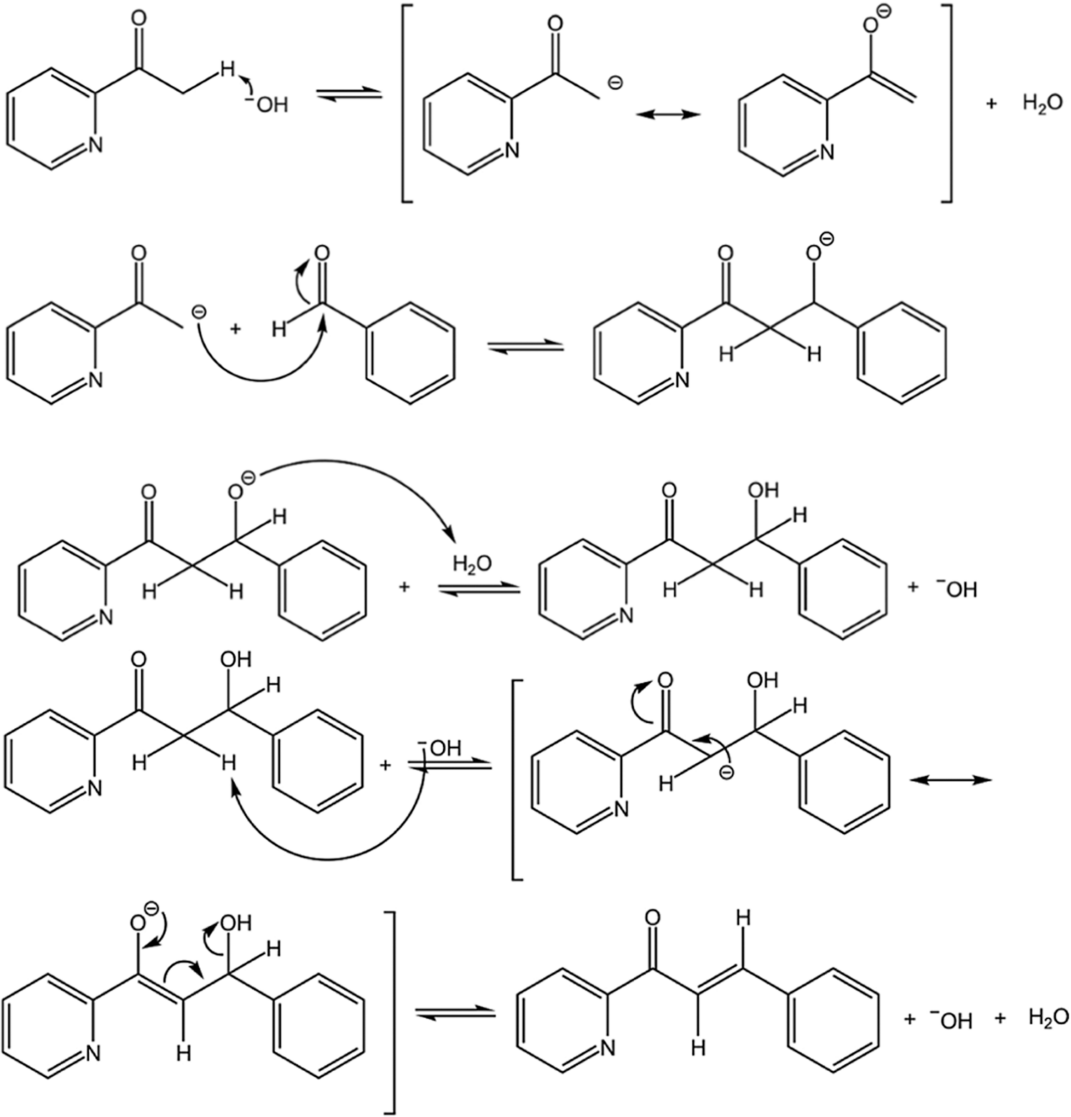
Route of the azachalcone synthesis.

**Figure 3:**
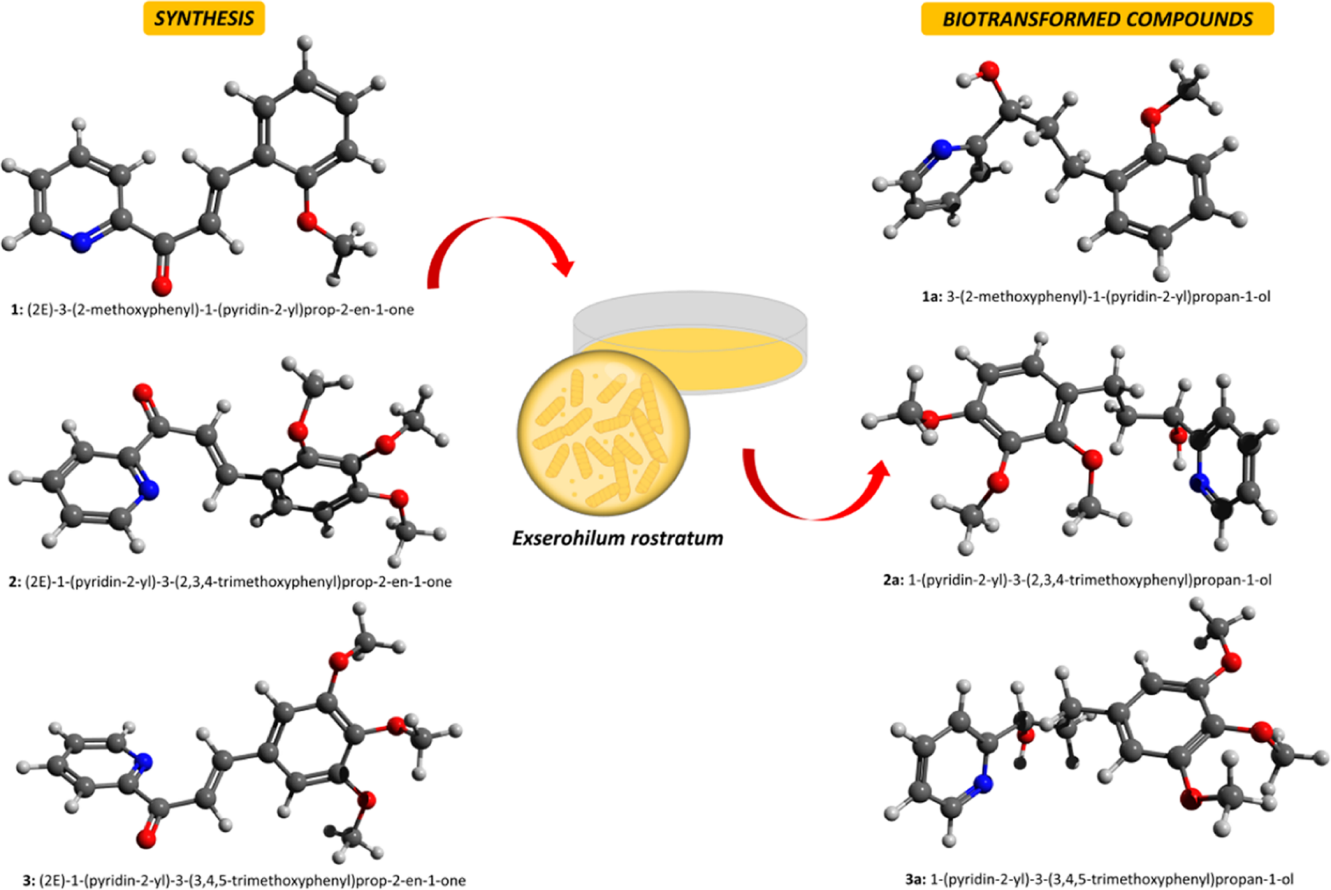
Structural representation of the compounds under study. The chemical elements are colored white (hydrogen), gray (carbon), red (oxygen), and blue (nitrogen).

This investigation presents the synthesis of three azachalcones in Figure 1, along with their pharmacological and optical uses. These chromophores are taken by substituting benzene with a pyridine ring in the main chalcone backbone. Subsequently, these compounds are biotransformed by an enzymatic reaction using the *Exserohilum rostratum* fungus, producing new compounds (see Figures 2 and 3, right side). The antimicrobial efficacy of these molecules, as evidenced by *in vitro* assays, opens possibilities for their development as novel pharmacological agents.

The NLO behavior of these chromophores was investigated using Density Functional Theory (DFT) calculations.^9,10^ The synthesized chromophore exhibited the highest NLO response, characterized by large first and second hyperpolarizabilities (β and γ). Conversely, the biotransformed compounds displayed the lowest refractive index (*n*), making them attractive candidates for light-conducting applications. These results, coupled with the antimicrobial potential, highlight the promising multifunctional nature of these chromophores for both biological and NLO applications.

## 2 Methodology

### 2.1 Experimental Details

#### 2.1.1 Chemical Synthesis

Azachalcones were obtained by the Claisen-Schmidt condensation reaction between a substituted benzaldehyde and 2-acetyl-pyridine using the methodology described in Corrêa et al.^11^ In an ice bath, an Erlenmeyer flask (125 mL), 15 mL of EtOH, 11 mmol of 2-acetyl-pyridine, 15 mL of 10% NaOH solution, and 12 mmol of substituted benzaldehyde were added. The reaction mixture was left under magnetic stirring at 40 °C for 3 h.

After this period, the mixture was cooled, left in a freezer for 48 h, and then vacuum filtered. The obtained product was recrystallized from methanol, and further identification analyses and yield calculations were performed. The obtained product was purified by elution of CC silica with hexane and ethyl acetate in a polarity gradient and identified by NMR and MS. These procedures were performed independently to yield each of compounds **1**, **2**, and **3**.

#### 2.1.2 Reaction of Biotransformation

First, the fungus *E. rostratum* was reactivated in a Petri dish containing a PDA culture medium at room temperature for 7 days. Then, for each of the azachalcones individually (**1**, **2** and **3**), the following procedure was carried out: six 500 mL Erlenmeyer flasks were individually sterilized with 250 mL Czapeck culture medium in a 75 L vertical autoclave (Prismatec) for 20 min at 121 °C. After reaching room temperature, in a laminar flow hood (Panchane PA 320), two small 2 mm^3^ cubes of the fungus were added into five flasks. Three flasks were used as controls (one flask containing fungus plus culture medium (MF), another flask containing culture medium plus substrate (MS), and another flask containing only culture medium (M)) and three flasks for bioreduction.

The system was mechanically shaken in an orbital shaker (Quimis Q315IA) at 120 rpm at a controlled temperature of 32 °C for the growth of fungal colonies for 3 days. Then, 40 mg (per flask) of each substrate was solubilized in 100 μL of DMSO and added to three Erlenmeyer flasks. The system was kept under agitation and formation of the products for 3 days. Then, the flasks were removed and filtered, and the extracellular liquid medium was extracted with ethyl acetate (3 × 75 mL). The obtained ethyl acetate solution was concentrated in a rotary evaporator to obtain the bioreduction products. The reactions of bioreduction showed yields of 10% for **1a**, 50% for **2a**, and 15% for **3a**.

#### 2.1.3 Antimicrobial Assays

Test microorganisms tested were *Bacillus subtilis* (ATCC 6633), *Escherichia coli* (ATCC 25922), *Staphylococcus aureus* (ATCC 25923), and *Salmonella typhimurium* (ATCC14028), which were obtained from Instituto Evandro Chagas, Belém, PA, Brazil. These assays were performed by applying the broth microdilution method according to the standards described by the Clinical and Laboratory Standards Institute (CLSI, 2017). In 96 wells, we added 100 μL of culture medium brain heart infusion (BHI) (Himedia), 100 μL of test material, and 5 μL of test bacteria at 1.0 × 10^6^ CFU mL^–1^, which were incubated at 37 °C (24 h).

The compounds obtained were dissolved initially in 1 mg of dimethyl sulfoxide in 100 μL of dimethyl sulfoxide and 900 μL of BHI broth given 1 mg mL^–1^ for stock solution. The stock solution was diluted to 250 to 3.91 μg mL^–1^. Bioactivity was recorded as an absence of red coloration in the wells after adding 10 μ L of 2,3,5-triphenyltetrazolium chloride. Amoxicillin and tetracycline were employed as positive controls, and BHI culture medium was used as a negative control.

### 2.2 General Procedure

1D and 2D nuclear magnetic resonance spectra were recorded on a Bruker Ascend 400, using the solvent signal (d-chloroform) as a reference. Chemical shifts are expressed in delta values (δ) and coupling constants (*J*) in hertz (Hz). Mass spectral data (ESIMS) were acquired using a UHPLC-MS/MS - Thermo Q Exactive Orbitrap mass spectrometer.

### 2.3 Theoretical Details

This investigation performs three kinds of electronic calculations within the DFT framework using the integral-equation formalism of the polarizable-continuum model (IEF-PCM)^12^ simulating the liquid environment. First, geometry optimization and the analysis of the infrared spectra confirmed the absence of negative vibrational frequencies and, consequently, the presence of minimal energy.

Subsequently, calculations of nuclear magnetic resonance (NMR) within the Gauge-Independent Atomic Orbital formalism (GIAO)^13^ provide an experiment-theory comparison, aiming to confirm the structures of the primary and secondary metabolites.

Electrical linear and NLO parameters are also the focus of this investigation. These effects arise when light interacts with matter. In such a case, the energy of the system can be expanded in a Taylor series like
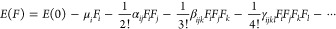
1

In this equation, μ is the permanent dipole moment, α is the dipole polarizability, a tensor of rank 2, which the diagonal components might be combined to give the isotropic contribution (α_iso_)
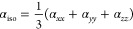
2

The isotropic polarizability can be used to infer the refractive index (*n*) using the Lorentz–Lorenz equation^14,15^
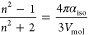
3where *V*_mol_ is the molecular volume.

Concerning the first hyperpolarizability (β), it is a 3 × 3 × 3 tensor with 27 components, and in the presence of frequency-dependent light, this quantity is better described by the Hyper-Rayleigh scattering (HRS) apparatus^16,17^ to give the frequency-dependent first hyperpolarizability as
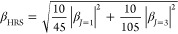
4

In this equation, β_*J*=1_ and β_*J*=3_ correspond to the dipolar and octupolar tensor, respectively written like
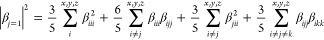
5and
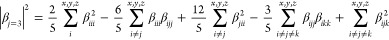
6

These two tensors can be combined to give the anisotropy factor , which defines the dipolar , and octupolar  contributions. Alternatively, one can discuss the dipolar-octupolar relationship introducing the concept of depolarization ratio, ,^16,17^ which might be obtained manipulating the tensorial components of β_HRS_.

The second hyperpolarizability (γ) is the last parameter of interest. It is responsible for the third harmonic generation effect. This one is a fourth-rank tensor (3 × 3 × 3 × 3) with 81 components. Fortunately, within the Electric-Field-Induced Second-Harmonic Generation setup, the middle indexes can be interchanged γ_*ijkl*_ = γ_*ikjl*_. Such simplification allows us to work with only 15 components, and the third harmonic generation γ(−3ω;ω,ω,0) = γ_THG_ is given as^18^
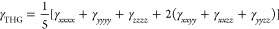
7

All quantum mechanical calculations were performed using the M06-2X density functional^19^ with the 6-311++G(*d,p*) basis set,^20,21^ as recently recommended for NLO^22^ and NMR^23^ properties. Calculations were carried out using the Gaussian 09 program,^24^ and NLO properties were analyzed with the Multwfn package.^25^

## 3 Results and Discussion

### 3.1 Molecular Characterization

For azachalcones **1**, **2** and **3**, the ^1^H NMR spectra (400 MHz; CDCl_3_), the signals related to hydrogens H-8 and H-9 were identified as an **AB** type system at 7.87–8.34 ppm, as a doublet of coupling constant of *J* ≈ 16 Hz characterizing the *trans* conformation of the chalcones, as well as the signals related to the aromatic hydrogens and those bonded to the methoxyl hydrogens. In the **B** carbon ring, the AA′BB′ system of H-3′-H-5′ and H-2′-H-6′ was verified, with the relative signals between 6.70 and 7.80 ppm. The hydrogens H-3, H-4, H-5, and H-6 in the **A** ring are structurally equivalent for the chalcones **1**, **2**, and **3** with signals observed at 7.45–8.76 ppm (see Table 1).^26−28^

**Table 1:** Experimental ^1^H and ^13^C NMR Data to Compounds **2** and **2a** (400 MHz, CDCl_3_) and Theoretical Chemical Shifts Using the Calculation Level M06-2*X*/6-31+G*, with the Simulations Performed in Solvent (Chloroform) by the IEF-PCM and GIAO Methods^a^

	**2 (synthesis)**				**2a (new compound)**			
n°	^1^H		^13^C		^1^H		^13^C	
	δ (exp.)	δ (theor.)	δ (exp.)	δ (theor.)	δ (exp.)	δ (theor.)	δ (exp.)	δ (theor.)
**1**			154.6				162.2	
**2**								
**3**	8.73 (d, 4.7)	9.19	148.8	146.85	8.54 (d, 4.8)	9.35	148.1	149.33
**4**	7.45 (dd, 6.0, 5.7)	7.78	126.6	124.20	7.19 (dd, 5.4, 5.1)	7.82	122.2	121.01
**5**	7.85 (t, 7.8)	8.27	137.0	138.12	7.68 (t, 7.8)	8.15	136.7	137.06
**6**	8.17 (d, 7.8)	8.10	122.8	125.55	7.32 (d, 7.8)	7.60	120.4	121.71
**7**		89.5	191.21	4.75 (dd, 8.2, 3.8)	4.36		72.2	62.74
**8**	8.18 (d, 16.5)	7.53	121.1	124.10	1.94 (m), 2.10 (m)	1.85, 1.32	39.5	34.99
**9**	8.23 (d, 16.5)	8.55	139.8	151.29	2.72 (m)	1.78, 2.51	25.6	16.60
**1′**			122.4	117.81			127.8	131.44
**2′**			154.0	151.55			151.8	148.17
**3′**			142.4	137.48			142.3	141.83
**4′**			155.9	153.24			152.0	150.46
**5′**	6.71 (d, 8.9)	6.86	107.6	100.82	6.60 (d, 8.6)	7.14	107.4	114.66
**6′**	7.55 (d, 8.9)	7.25	123.6	131.27	6.87 (d, 8.6)	6.71	124.1	124.14
**OMe-2′**	3.99	4.04, 4.66, 3.22	61.7	48.97	3.86 (s)	2.94, 4.22, 2.79	61.0	50.22
**OMe-3′**	3.91	2.97, 5.07, 3.83	60.9	49.18	3.85 (s)	3.61, 2.94, 4.22	56.0	46.22
**OMe-4′**	3.94	4.29, 3.61, 3.53	56.1	43.49	3.83 (s)	3.61, 3.97, 2.94	60.7	46.52

a Legend: H: Hydrogen; C: Carbon; δ: Chemical shift (ppm).

The products of bioreduction of the dihydroazachalcones were characterized by ^1^H NMR through the absence of the signals for the trans-H-8/H-9 system and the signals for the olefinic carbons C-8/C-9 in ^13^C NMR. The appearance of the signals for oxymethynic hydrogens at around 4.80 ppm in ^1^H NMR for an oxygenated carbon at around 72.0 ppm in ^13^C NMR confirms the bioreduction. HSQC and HMBC correlations allowed complete identification of the compounds **1a**, **2a**, and **3a**.^27^ The compound **2a** is new in the literature, and its structure was confirmed by extensive analysis of NMR data and HRMS with *m*/*z* = 304.1552 [M + H] (Supporting Information, Figures S1–S15).

### 3.2 Antimicrobial Response and Possible Structure–Activity Relationship

All tested compounds exhibited significant antimicrobial activity (see Table 2), with **1** and **1a** demonstrating the most potent inhibition against the Gram-negative bacterium *S. typhimurium*, achieving complete inhibition at the lowest tested concentration of 3.91 μg/mL. The bioreduction of **2** to **2a** and **3** to **3a** significantly enhanced antibacterial activity, particularly against *E. coli*, with a 4-fold and thirty-fold increase, respectively.

**Table 2:** In Vitro Minimum Inhibitory Concentration (MIC, μg/mL) of Compounds against Gram-Positive and Gram-Negative Bacteria

	bacteria (MIC, μg/mL)			
	Gram-positive bacteria		Gram-negative bacteria	
compounds	*B. subtilis*	*S. aureus*	*S. typhimurium*	*E. coli*
**1**	7.81	250	3.91	250
**1a**	7.81	250	3.91	250
**2**	7.81	250	31.25	250
**2a**	250	250	250	62.25
**3**	7.81	250	125	250
**3a**	15.62	250	250	7.81
**tetracycline**	3.91	3.91	3.91	3.91
**amoxicillin**	3.91	3.91	3.91	3.91

The synthesized azachalcones possess two aromatic rings linked by an α,β-unsaturated carbonyl system with trans-related H-8 and H-9 hydrogens. Fungal biotransformation by *E. rostratum* reduced the HC = CH and C = O double bonds, disrupting conjugation and increasing molecular flexibility due to greater rotational freedom around the C-8 and C-9 bonds. This reduction significantly altered the compounds’ activity, suggesting that the enhanced activity is primarily attributed to interactions between the aromatic rings rather than the α,β-unsaturated carbonyl system. The increased conformational flexibility likely facilitates better binding to enzymes crucial for bacterial survival, leading to an increased efficacy.

While a slight improvement in activity against Gram-positive bacteria was also observed, the thicker outer membrane of Gram-positive bacteria might hinder the penetration of antibacterial agents. However, the agents may still damage the membrane, causing a bactericidal effect, as suggested by previous studies.^29,30^

### 3.3 Dipole Moment

The dipole moment (μ) is an electrical property that determines, on average, the distribution of electrical charges and molecular shape. In addition, molecular dipole moments are important for many reasons, such as the ability to dissolve solutes, melting and boiling points, and overall reactivity.

Table 3 presents the obtained values for the permanent dipole moment of the studied compounds calculated in an aqueous environment using the quantum mechanical M06-2*X*/6-311++G(*d,p*) level and IEF-PCM as a solvent model. As can be seen, azachalcones from **1** to **3** have the most significant contributions and, consequently, better stabilization in polar solvents.

**Table 3:** Permanent Dipole Moment (μ/D), Dipolar Polarizability (α/10^–26^ esu), Molecular Volume (*V*_mol_/Bohr^3^·mol^–1^), and the Refractive Index (*n*) Calculated Using the M06-2*X*/6-311++G(*d,p*) in Water Using the IEF-PCM as Solvation Model

chromophore	μ	α_iso_	*V*_m_	*n*
**1**	8.44	39.18	1509.03	3.04
**1a**	3.84	35.94	2787.09	1.65
**2**	7.85	47.04	2100.10	2.48
**2a**	4.25	42.06	2421.48	1.97
**3**	2.50	46.14	2864.54	1.87
**3a**	3.34	42.20	2134.61	2.19

On the other hand, substituting HC = CH by H_2_C–CH_2_ drastically affects the permanent dipole moment. Compared with the synthesis, the new compounds show a drastic reduction in this property. For example, the chromophore **1a** has a value of 3.84 D, which is a reduction of 119%. A similar trend can be observed for compound **2a**, which exhibits a dipole moment of 4.25 D. However, with a dipole moment of 3.34 D, chromophore **3a** is the only one that increases compared to its synthesis.

### 3.4 Dipolar Polarizability and Refractive Index

Regarding the isotropic contribution to the dipolar polarizability (α_iso_), the highest values were recorded for the synthesis (**1** to **3**). According to the M06-2*X*/6-311++G(*d,p*) performed in water solvent using IEF-PCM, for example, α_iso_ varies from 39.18 × 10^–26^ to 47.04 × 10^–26^ esu. On the other hand, the chromophores **1a** to **3a** present values between 35.94 × 10^–26^ to 42.20 × 10^–26^ esu for α_iso_.

Knowledge of dipole polarizability (α) is essential for understanding intermolecular interactions, NLO, and the design of organic electro-optical (OEO) materials, to name a few areas. In optics, if the molecular volume (*V*_mol_) is known, it is possible to connect the polarizability of the dipole (α_iso_) to the refractive index (*n*) by the Lorentz–Lorenz equation and, consequently, predict how a chromophore would transmit light. Physically, the lower the refractive index, the better the information transmission. According to Table 3, the azachalcones **1a** and **3**, those with the lowest values of α_iso_ relate the best refractive index, 1.65 and 1.87, respectively.

However, compared with other dyes specially designed for NLO uses, biotransformed azachalcones exhibit competitive refractive index values. For example, Raiol and co-workers have investigated the optical behavior and *cis–trans* isomerization effect of some large azomethine dyes specially developed for optical applications. The authors obtained refractive indices ranging from 1.758 to 2.374 for 1-nitro-2-phenylethane in the aqueous solvent.^31^

### 3.5 First Hyperpolarizability

Table 4 presents results for the first frequency-dependent hyperpolarizabilities obtained according to X-ray scattering (β_HRS_). Among the syntheses, **1** is the one that presents the OCH_3_ group in the ortho position, and the lowest value for the first hyperpolarizability, 14.54 × 10^–30^ esu. However, from chromophore **1** to **2**, the largest difference is the number of methoxy groups. In this case, β_HRS_ becomes 34.73 × 10^–30^ esu, which means an increase of more than 115% (see Table 4). These results are consistent with previous works that predict that the methoxy group increases the NLO response.^32^

**Table 4:** First hyperpolarizability (β_HRS_/10^–30^ esu), Dipolar and Octupolar Contributions (Φ_*J*=1_ and Φ_*J*=3_), Depolarization and Anisotropy Ratios (DR and ρ), the Second Hyperpolarizability (γ_THG_/10^–34^ esu) and the Energy Gap (Δ*E*_g_/eV) Calculated Using M06-2*X*/6-311++G(*d,p)* in Water Using the IEF-PCM as a Solvation Model

chromophore	β_HRS_	Φ_*J*=1_	Φ_*J*=3_	ρ	DR	γ_THG_	Δ*E*_g_
**1**	14.54	0.513	0.487	0.948	4.45	0.99	6.25
**1a**	2.07	0.703	0.297	0.422	7.25	0.32	7.37
**2**	34.73	0.532	0.468	0.881	4.71	2.26	7.48
**2a**	0.86	0.573	0.427	0.744	5.35	0.37	7.52
**3**	27.37	0.555	0.445	0.801	5.07	1.99	5.96
**3a**	2.13	0.396	0.604	1.525	3.00	0.38	7.37

Chromophore **3** has three methoxy groups at ortho, meta, and para positions. Under this configuration, the highest NLO behavior is observed with β_HRS_ = 34.73 × 10^–30^ esu. On the other hand, chromophore **3** exhibited a slightly lower optical coefficient value.

The above results for the proposed aza dyes are superior to those of other standard optical materials. For example, for *p*-nitroaniline, DFT results indicate first hyperpolarizabilities ranging from 6.27 × 10^–30^ esu^33^ and 8.86 × 10^–30^ esu,^34^ which are almost four times lower than those reported for current azachalcones. More pronounced is the case for urea, another common NLO material, which has an optical coefficient approximately one hundred times lower (β = 0.34 × 10^–30^ esu).^35^

**Figure 4:**
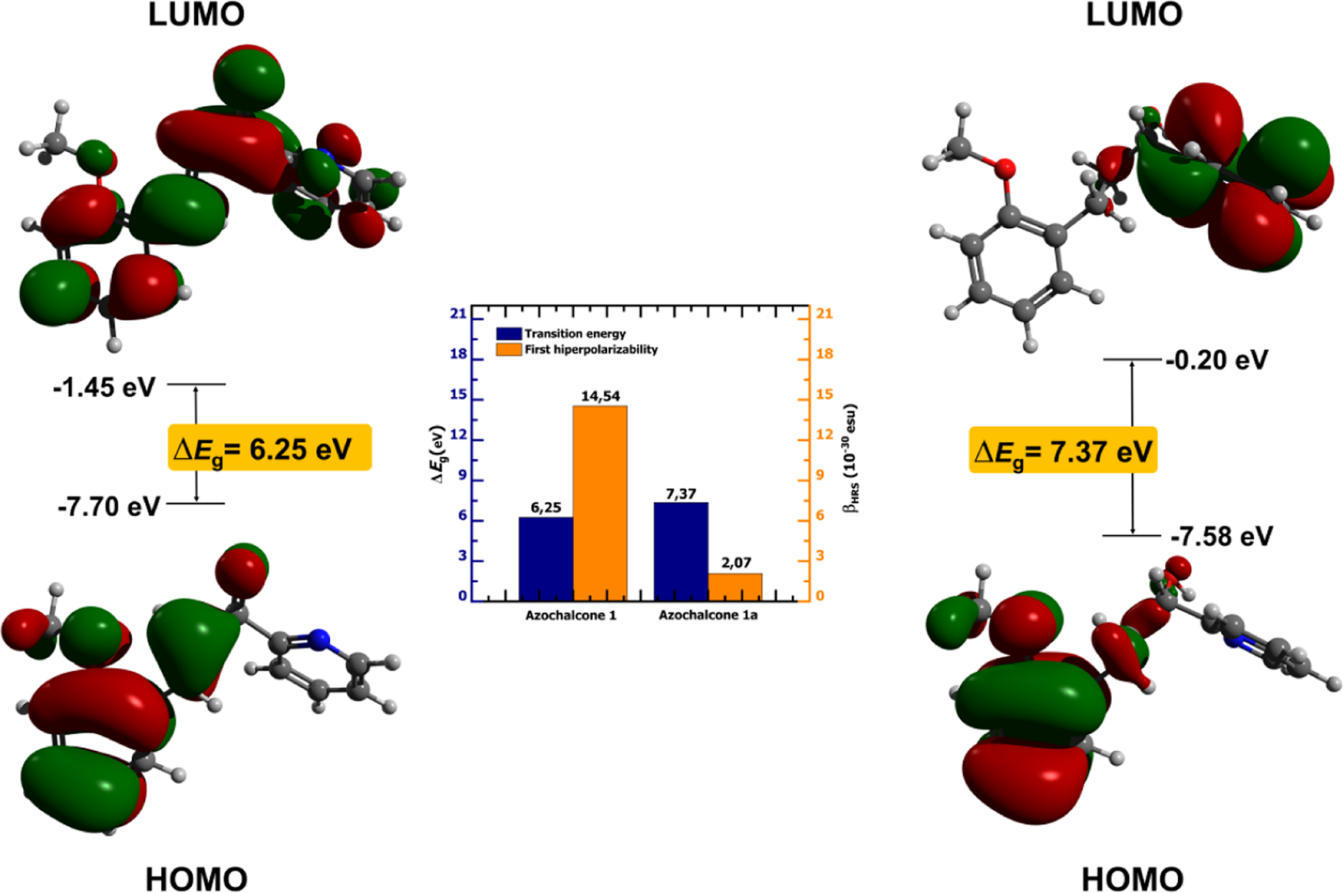
Highest occupied and lowest unoccupied molecular orbitals (HOMO and LUMO) plotted for **1** (left side) and **1a** (right side) using the M06-2*X*/6-311++G(*d,p*) level of quantum mechanics.

As for the effect of the biotransformation reaction, a significant decrease in the NLO behavior of the chromophores can be observed. For example, Table 4 indicates that chromophores **1a**–**3a** show first hyperpolarizabilities ranging from 0.86 × 10^–30^ to 2.13 × 10^–30^ esu. This effect occurs due to the substitution of HC = CH with H_2_C–CH_2_, which significantly affects the energy levels of the material. For example, in Figure 4, it can be seen that the frontier molecular orbitals of **1** are delocalized along the molecule, evidencing an overlap that facilitates electronic excitation.^36^ A similar behavior is found for azachalconas **2** and **3** (see Supporting Information, Figures S16–S18).

However, for syntheses such as **1a**, it is observed that the molecular orbitals are located in different molecular regions. This condition increases the energy gap (Δ*E*_g_) from 5.96 to 7.37 eV. The problem is that according to the two-level quantum mechanical description proposed by Oudar and Chemla,^37^ β decays with the third power order of Δ*E*_g_ obeying a relation like
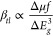
8

Figure 4 expresses exactly this behavior between β_HRS_ and Δ*E*_g_ for **1** and **1a**. Moreover, a quick look at Table 4 shows that all syntheses and new compounds retain this relationship.

### 3.6 Dipolar and Octupolar Contributions to the NLO Response

The NLO behavior of a chromophore consists of dipolar (Φ_*J*=1_) and octupolar (Φ_*J*=3_) contributions that can be combined to give the anisotropy ratio (ρ), which ranges from completely dipolar behavior if ρ → 0 and octupolar behavior if ρ → ∞. Since different optoelectronic devices can be proposed depending on the nature of the NLO response, it is crucial to classify a compound as a dipolar, intermediate, or even octupolar chromophore.

**Figure 5:**
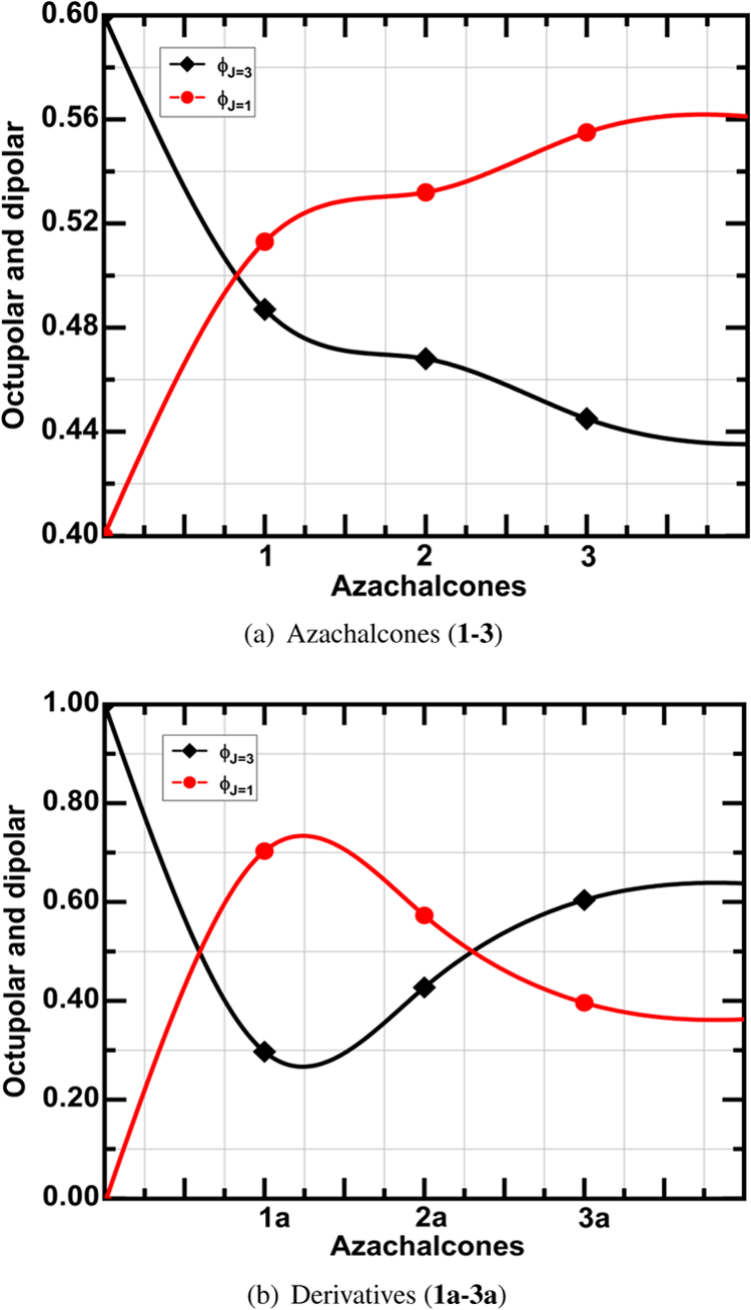
Octupolar (Φ_*J*=3_) and dipolar (Φ_*J*=1_) contributions plotted the (a) azachalcones (**1**–**3**) and their (b) derivatives (**1a**–**3a**).

According to Table 4, all chalcones (**1**–**3**) exhibit lower values of anisotropy ratio (ρ < 1), indicating predominant dipolar characteristics. Figure 5 better shows this behavior by tracing the evolution of the octupolar and dipolar contributions. From **1** to **3**, it is observed that the systematic inclusion of methoxy groups increases the dipolar contribution to the detriment of its symmetric counterpart. This behavior could be understood as a function of molecular geometry. In Figure 3, it can be seen that all synthesized chalcones are almost one-dimensional systems, favoring a one-dimensional charge transfer procedure, which is the main feature of the dipolar behavior of NLO.^36,38^

A different behavior is observed regarding the biotransformation effect that generated chromophores **1a**–**2a**. This time, the dipolar contribution decreases from **1a** to **3a**, showing an octupolar predominance. This effect occurs because the substitution of HC = CH by H_2_C–CH_2_ breaks the charge transfer procedures and allows geometrical redistribution, affecting the molecular symmetry. These effects are reported to affect NLO parameters such as the first hyperpolarization.^39^

The concept of the depolarization rate (DR) confirms the dipolar characteristic of the NLO response. As discussed by Champagne,^17,40^ the range parameter varies from 1.5 to 9 for pure octupolar to dipolar architectures. From Table 4, the polarization ratio of chalcone (4.45 ≤ DR ≤ 5.07) and its new compounds (3.00 ≤ DR ≤ 7.25) are observed. According to the dipolar-octupolar scale due to Zhang and co-workers,^16^ which analyzes all parameters Φ_*J*=1_, Φ_*J*=3_, and DR, the two sets of chromophores (**1**–**3** and **1a**–**3a**) are best classified as intermediate dipolar molecules.

### 3.7 Second Hyperpolarizability

Table 4 also shows the results for the second hyperpolarization, which drives the third harmonic generation effect. Again, the lowest optical behavior belongs to chromophore **1** with 0.99 × 10^–34^ esu. However, for **3**, the methoxy groups on the benzene ring and γ_THG_ increase to 1.99 × 10^–34^ esu, which means a further 80% enhancement.

Again, chromophore **2** shows a substantial optical response. For this system, calculations indicate, for example, the second hyperpolarizability of 2.26 × 10^–30^ esu, about 128% higher than **1**.

In contrast, the lowest values reported for the second hyperpolarizability are associated with compounds obtained from biotransformation. According to Table 4 and calculations performed in water solvent, chromophores **1a**–**3a** present a range of values that vary from 0.32 × 10^–34^ to 0.38 × 10^–34^ esu, showing mild changes.

However, compared to other NLO materials, these chromophores exhibit interesting THG behavior. For example, Adant and co-workers have reported static values of 0.39 × 10^–36^ and 0.45 × 10^–36^ esu for the second hyperpolarization in urea.^41^ In other words, even the less sensitive azachalcone shows values almost a hundred times higher than those found for urea, which is a well-stabilized NLO chromophore.

## 4 Conclusions

This work investigates the synthesis, NMR characterization, bioconversion, pharmacological activity, and NLO properties of azachalcones. After synthesis, these chromophores were bioconverted enzymatically using the fungus *Exserohilum rostratum,* yielding secondary metabolites characterized by the bioreduction of the HC = CH and C = O double bonds. Notably, one of these secondary metabolites, 1-(pyridin-2-yl)-3-(2,3,4-trimethoxyphenyl)propan-1-ol, named **2a**, is novel.

*In vitro* biological assays against Gram-positive and Gram-negative bacteria revealed that compounds **1** and **1a** exhibited the most potent activity against *S. typhimurium* compared with the antibiotics tetracycline and amoxicillin. Moreover, the structural modifications resulting from the bioreduction reaction significantly enhanced the antibacterial activity of compounds **2a** and **3a** against Gram-positive bacteria, increasing their inhibitory potency by factors of four and 30, respectively.

DFT-based calculations of NLO properties indicated that the bioconversion reaction decreased the dipolar polarizability (α) and refractive index (*n*), properties desirable for light-guiding applications. Conversely, the first and second hyperpolarizabilities (β and γ) were significantly higher in the primary metabolites, exceeding the value of urea, a well-established NLO material, by over a hundredfold. These promising NLO properties suggest potential applications of these chromophores in photonics and optoelectronics, particularly in the development of advanced nanostructured circuits.

## Supporting Information Available

Data pertaining to the characterization of compounds (NMR and HRMS) and computational data (PDF)
