# Supplementary material for: Synthesis, Biotransformation, Characterization, and DFT Study of Organic Azachalcone Dyes and Secondary Metabolites with Biological and Conformation Dependence of Dipolar-Octupolar NLO Responses
Source: ACS Omega. 2025 Mar 10;10(11):10962–71. doi: 10.1021/acsomega.4c09074 (PMC11947782; doi:10.1021/acsomega.4c09074)
Supplement: Supplementary file 1 — ao4c09074_si_001.pdf [file ao4c09074_si_001.pdf]

Synthesis, biotransformation, characterization, and DFT study of organic azachalcone dyes and secondary metabolites with biological and conformation dependence of dipolar-octupolar NLO responses

Victoria L. Ribeiro[a], Neidy S.S. dos Santos[b], Raira V.S. de Oliveira[b], Joselina A. Carvalho[b], Viviane V. Garcia[b], Hartmann J.S. Brito-Junior[a], Willibrodus Usfinit[a], Mayra Pinheiro[c], Taicia Fill[c], Rodrigo Gester[e,f], Patricio F. Provasi[g], Sylvio Canuto[f], Heriberto R. Bitencourt[d], Patricia S.B. Marinho[a], Andrey M.R. Marinho*[a]

[a]Programa de Pós-Graduação em Química, Universidade Federal do Pará, Rua Augusto Corrêa, 01 - Guamá, 66075-110, Belém, PA, Brazil.
[b]Programa de Pós-Graduação em Química, Universidade Federal do Sul e Sudeste do Pará, 68507-590, Marabá, PA, Brazil.
[c]Instituto de Química, Universidade de Campinas, 13083-970, Campinas, SP, Brazil.
[d]Programa de Pós-Graduação em Ciências Farmacêuticas, Universidade Federal do Pará, Rua Augusto Corrêa, 01 - Guamá, 66075-110, Belém, PA, Brazil.
[e]Faculdade de Física, Universidade Federal do Sul e Sudeste do Pará, 68507-590, Marabá, PA, Brazil.
[f]Instituto de Física, Universidade de São Paulo, Rua do Matão 1371, 05508-090, São Paulo, SP, Brazil.
[f]Department of Physics, IMIT, Northeastern University, CONICET, AV. Libertad 5500, W 3404 AAS Corrientes, Argentina.

Email address: andrey@ufpa.br (Andrey M.R. Marinho)

Table of Contents

Figure S1: 1H NMR spectrum to compound 1a (400 MHz, CDCl3)..S3
Figure S2: 13C NMR spectrum to compound 1a (100 MHz, CDCl3)..S3
Figure S3. HRMS ESI (+) spectrum of m/z 304.1332 [M+H]+ (compound 1a)....................................S4
Figure S4. 1H NMR spectrum to compound 2a (400 MHz, CDCl3)..S4
Figure S5: 1H NMR spectrum to compound 2a ampliation δ 6.00 to 8.60 region.................................S5
Figure S6: 1H NMR spectrum to compound 2a amplification δ 4.80 to 4.70; δ 3.90 to 3.80 and δ 2.80 to 1.80 regions...S5
Figure S7: 13C NMR spectrum to compound 2a (400 MHz, CDCl3)..S6
Figure S8. HMBC spectrum to compound 2a (400 MHz, CDCl3)...S6
Figure S9. HSQC spectrum to compound 2a (400 MHz, CDCl3)..S7
Figure S10. COSY spectrum to compound 2a (400 MHz, CDCl3)...S7
Figure S11. HRMS ESI (+) spectrum of m/z 304.1552 [M+H]+ (compound 2a)..................................S8
Figure S12. MS/MS spectrum of m/z 304.1552 [M+H]+ (compound 2a)...S8
Figure S13: 1H NMR spectrum to compound 3a (400 MHz, CDCl3)..S9
Figure S14: 13C NMR spectrum to compound 3a (100 MHz, CDCl3)..S9
Figure S15. HRMS ESI (+) spectrum of m/z 304.1545 [M+H]+ (compound 3a)................................S10
Figure S16. HOMO and LUMO for compounds 1 and 1a..S10
Figure S17. HOMO and LUMO for compounds 2 and 2a..S11
Figure S18. HOMO and LUMO for compounds 3 and 3a..S11

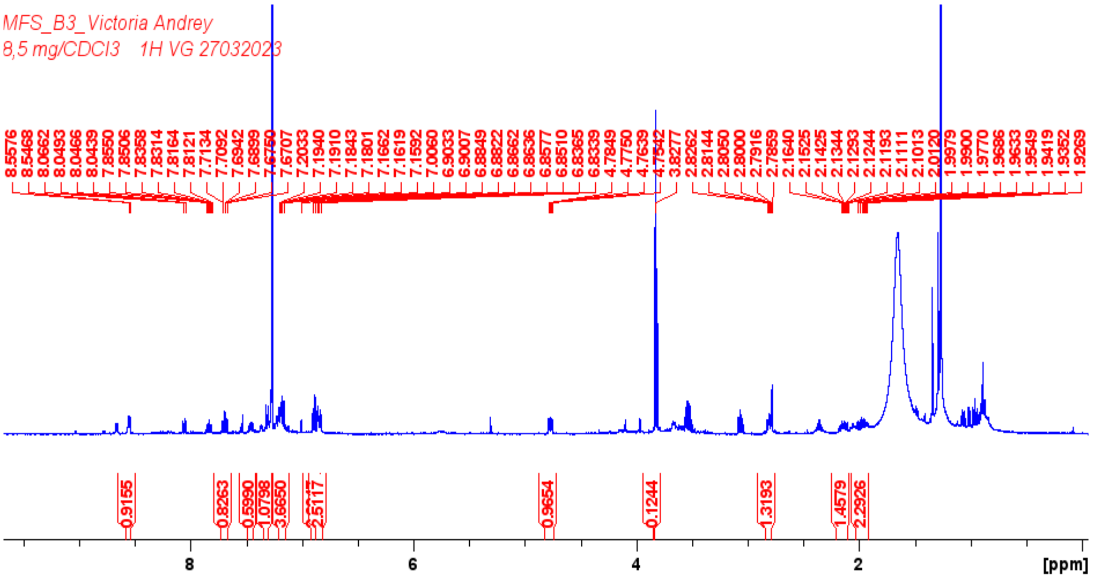

Figure S1: ^{1}H NMR spectrum to **compound 1a** (400 MHz, $CDCl_3$).

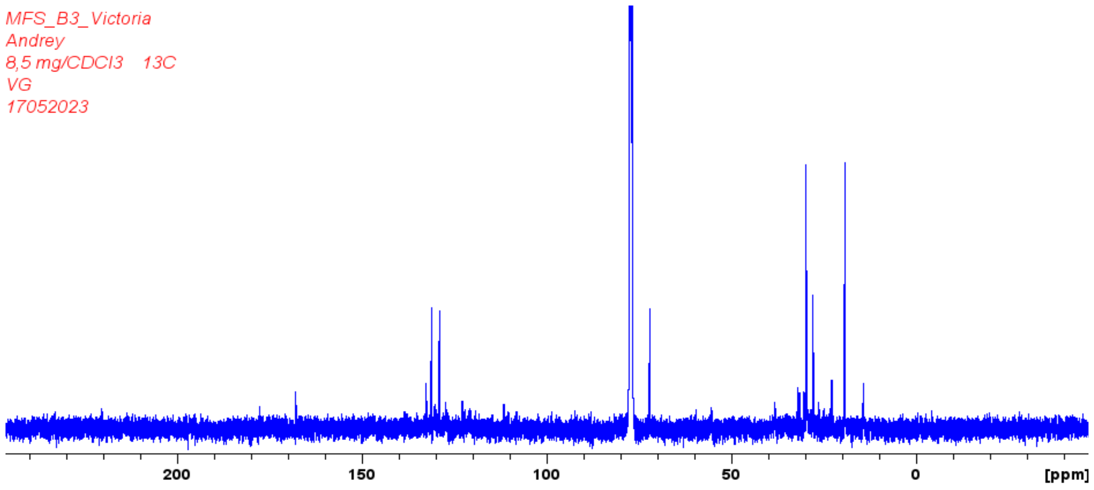

Figure S2: ^{13}C NMR spectrum to **compound 1a** (100 MHz, $CDCl_3$).

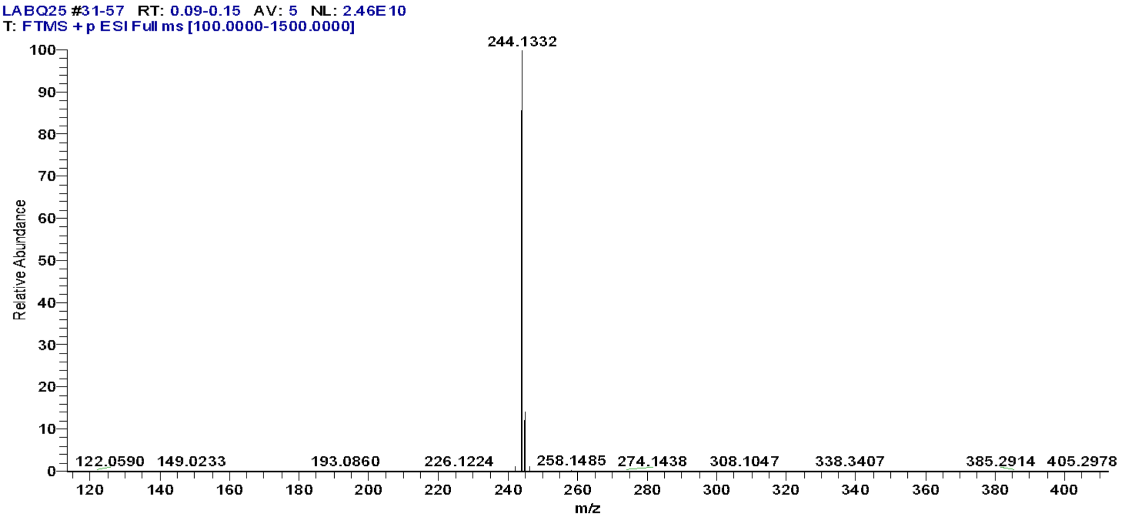

Figure S3. HRMS ESI (+) spectrum of *m/z* 304.1332 $[M+H]^+$ **(compound 1a)**.

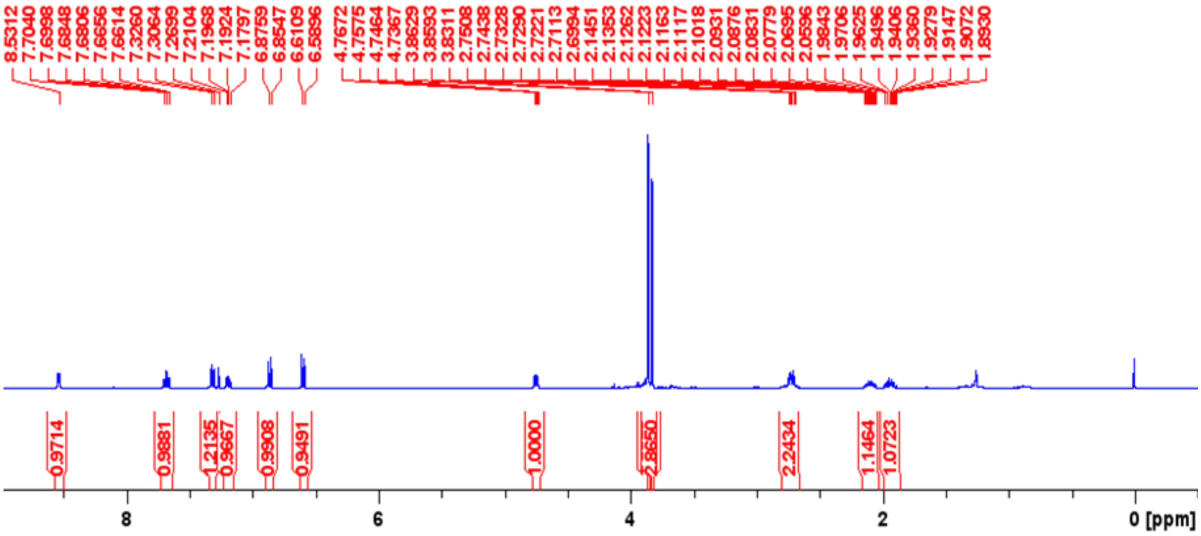

Figure S4. ^1H NMR spectrum to **compound 2a** (400 MHz, $CDCl_3$).

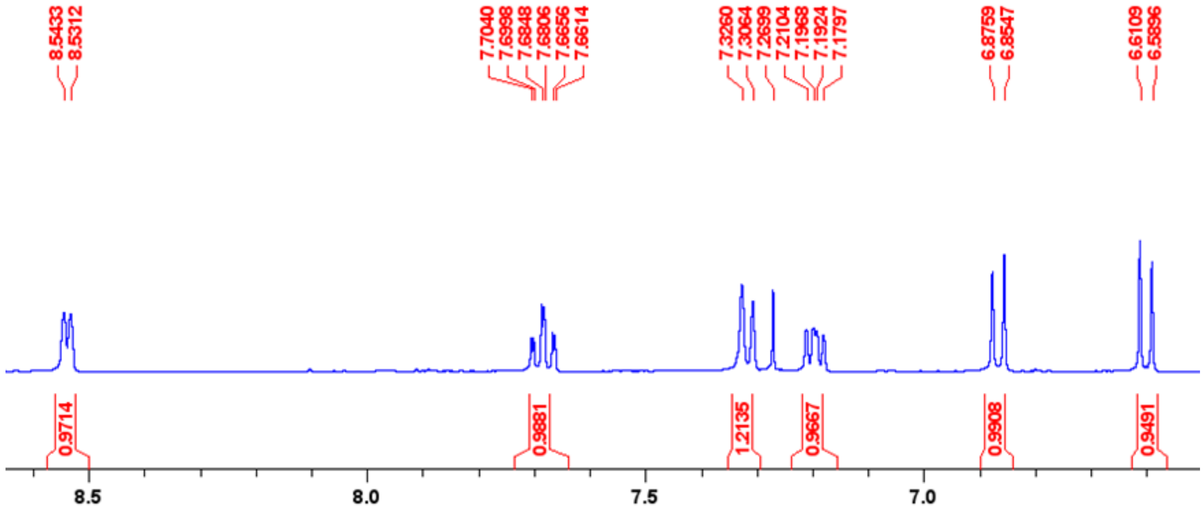

Figure S5: ^{1}H NMR spectrum to **compound 2a** ampliation δ 6.00 to 8.60 region.

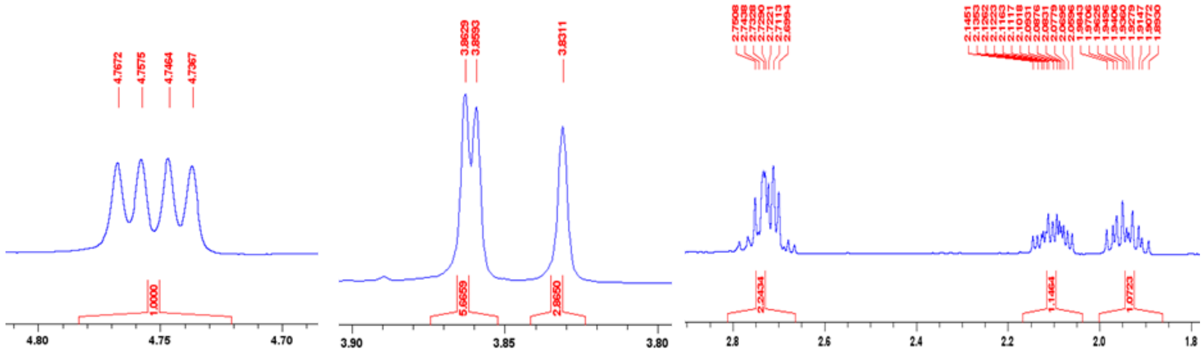

Figure S6: ^{1}H NMR spectrum to **compound 2a** amplification δ 4.80 to 4.70; δ 3.90 to 3.80 and δ 2.80 to 1.80 regions.

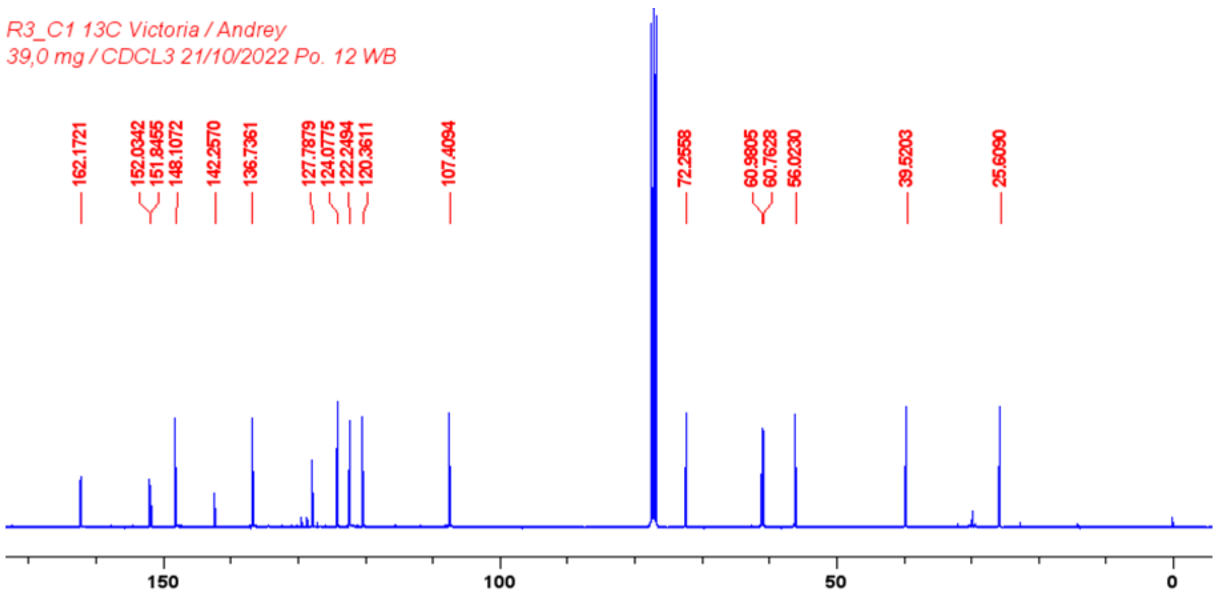

Figure S7: ^{13}C NMR spectrum to **compound 2a** (400 MHz, $CDCl_3$).

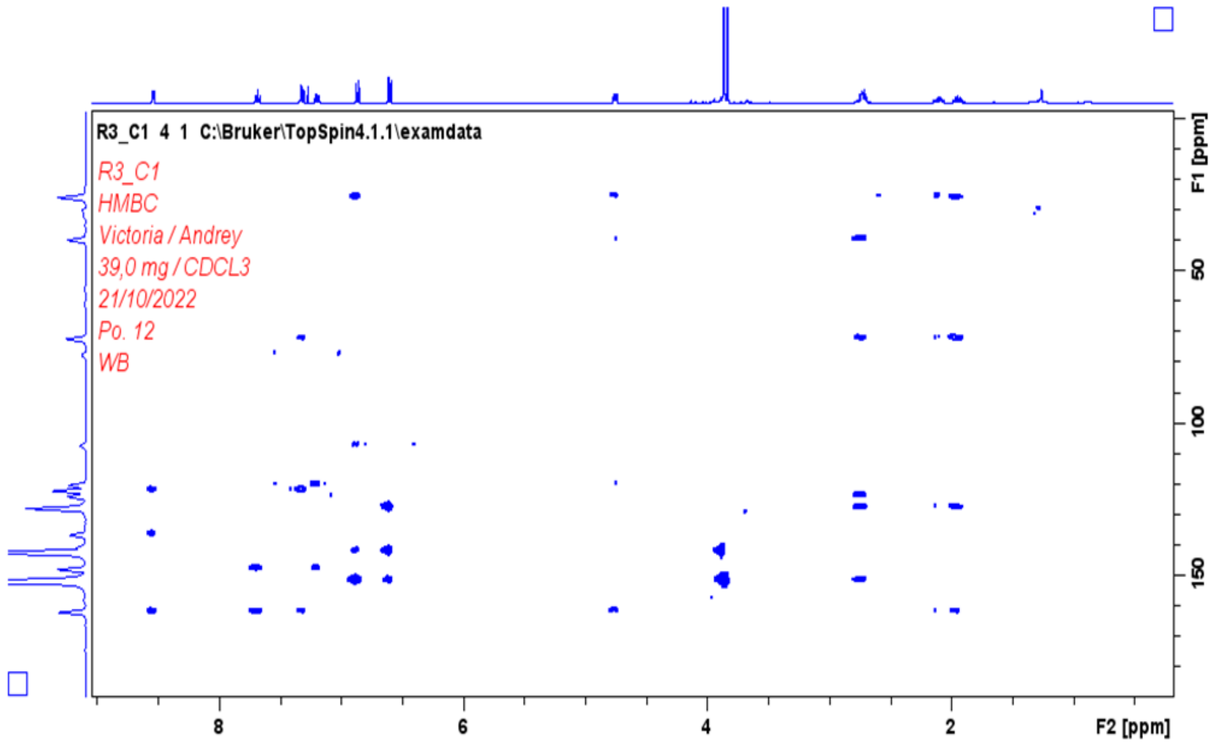

Figure S8. HMBC spectrum to **compound 2a** (400 MHz, $CDCl_3$).

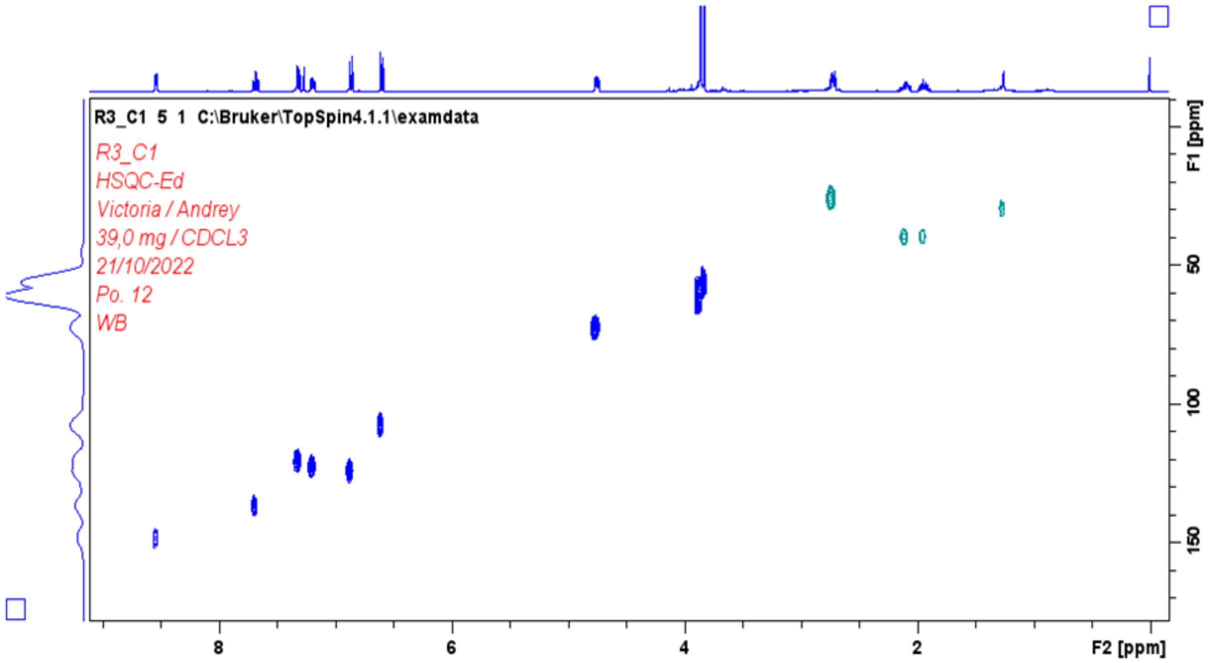

Figure S9. HSQC spectrum to **compound 2a** (400 MHz, $CDCl_3$).

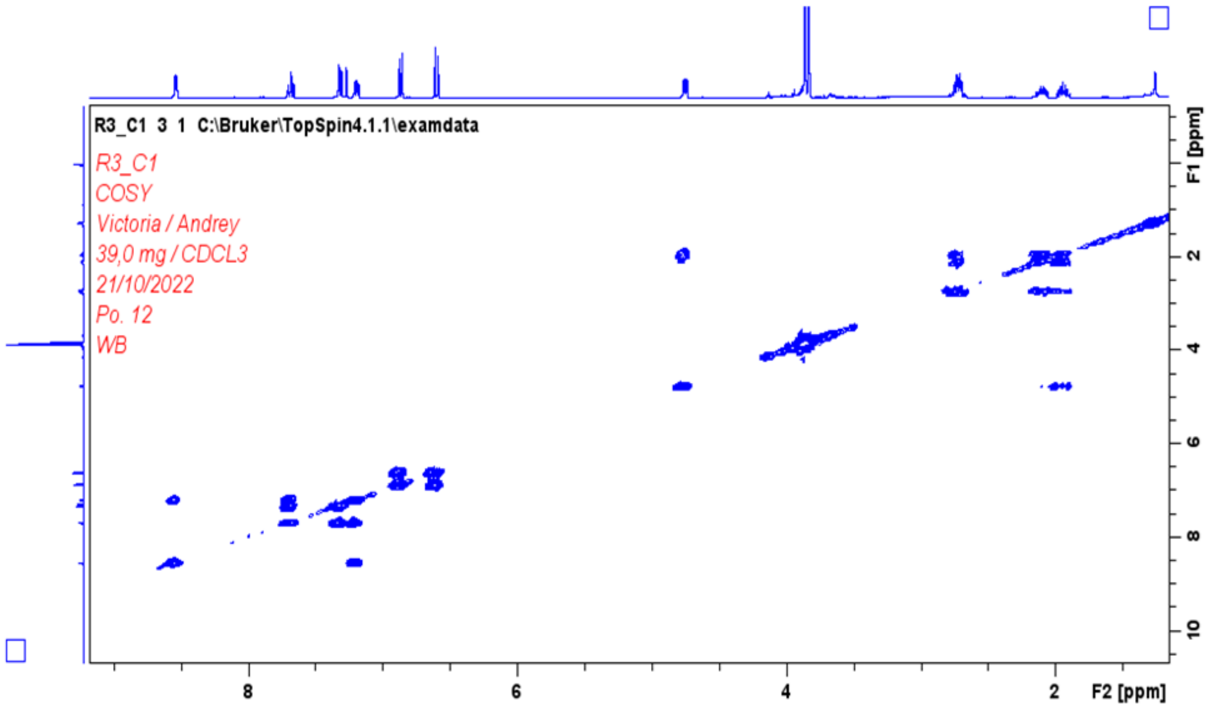

Figure S10. COSY spectrum to **compound 2a** (400 MHz, $CDCl_3$).

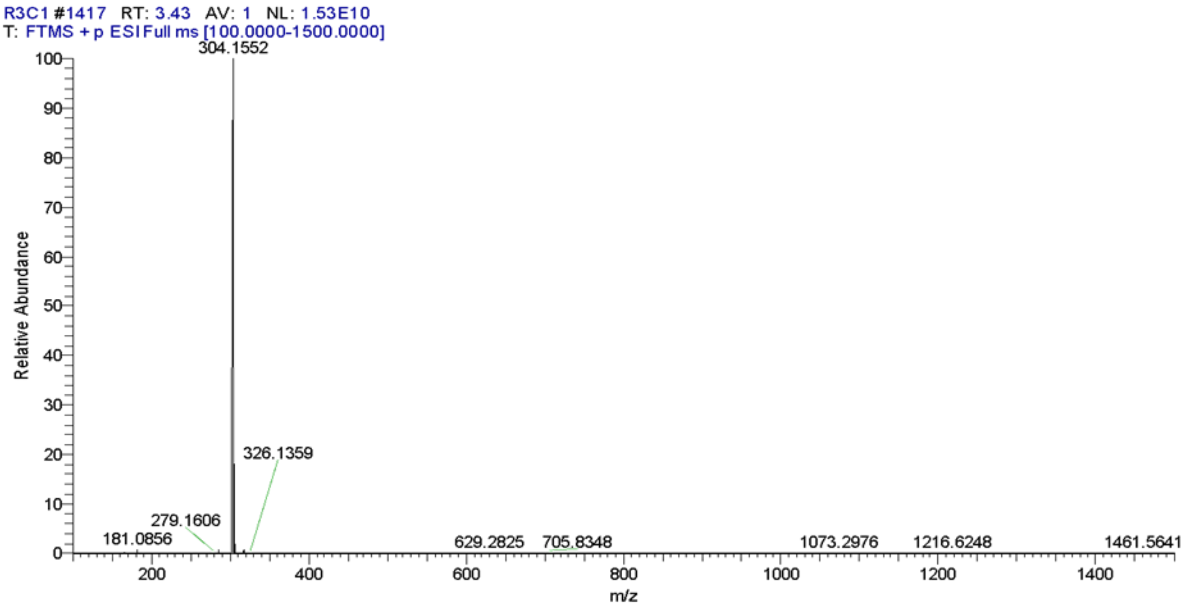

Figure S11. HRMS ESI (+) spectrum of *m/z* 304.1552 $[M+H]^+$ **(compound 2a)**.

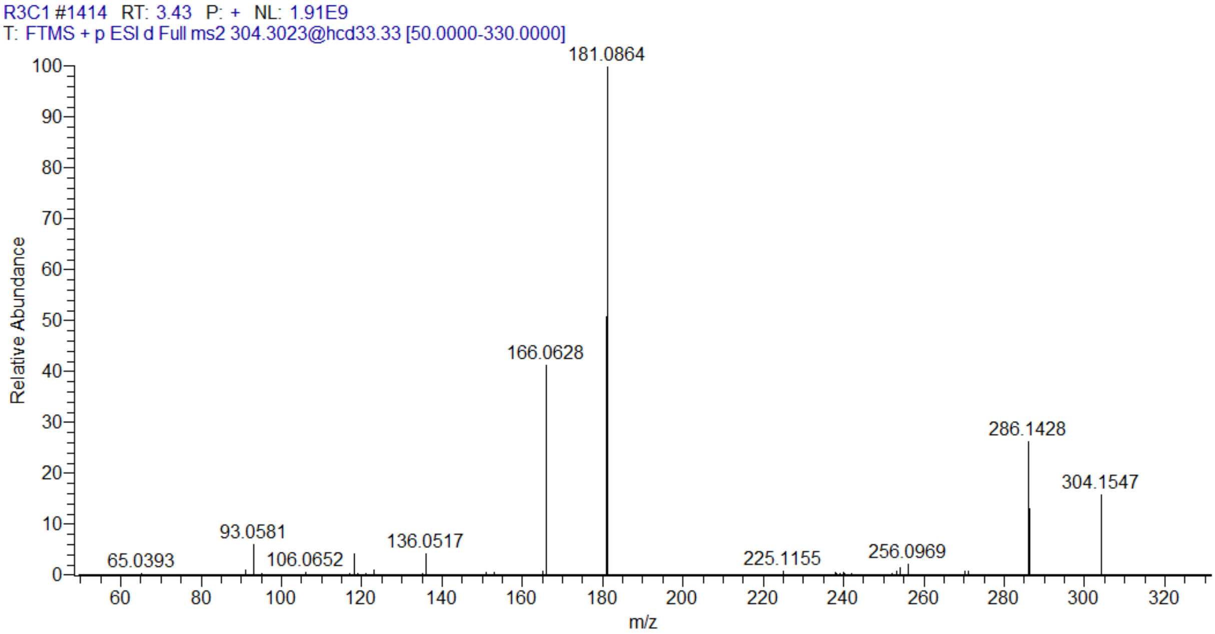

Figure S12. MS/MS spectrum of *m/z* 304.1552 $[M+H]^+$ (**compound 2a**).

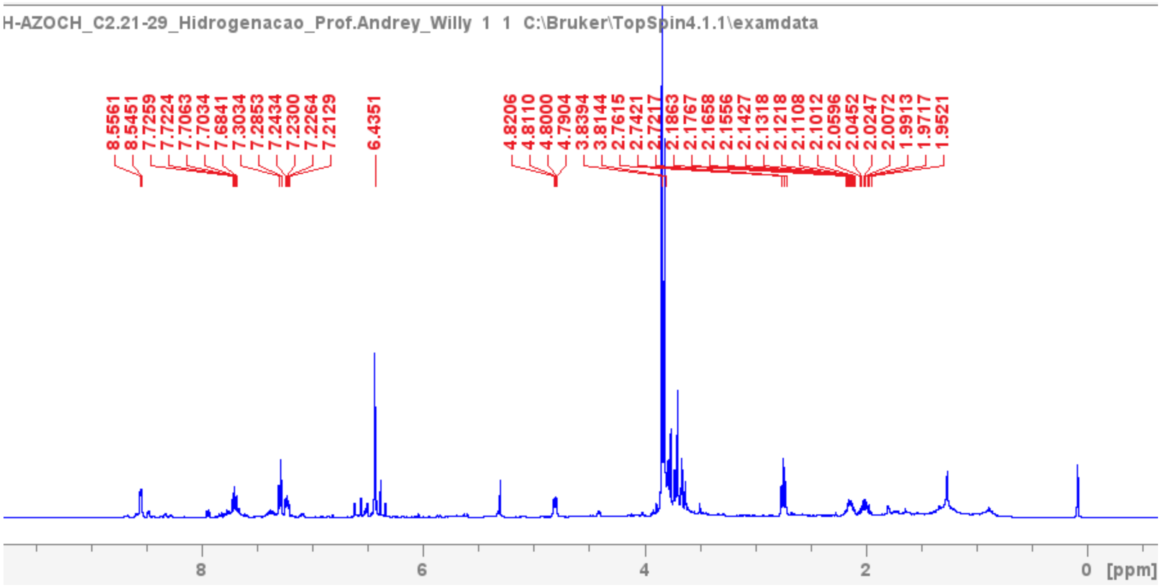

Figure S13: ^{1}H NMR spectrum to **compound 3a** (400 MHz, $CDCl_3$).

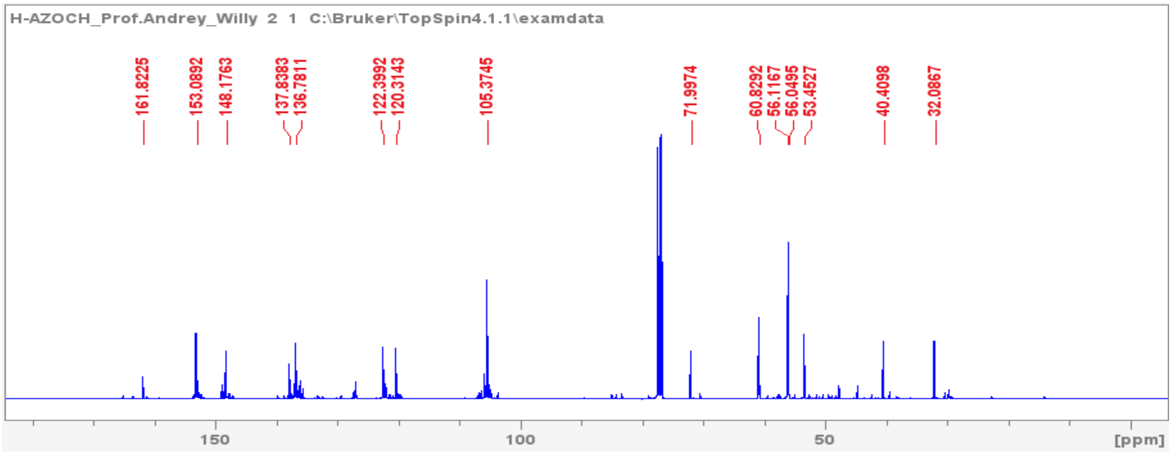

Figure S14: ^{13}C NMR spectrum to **compound 3a** (100 MHz, $CDCl_3$).

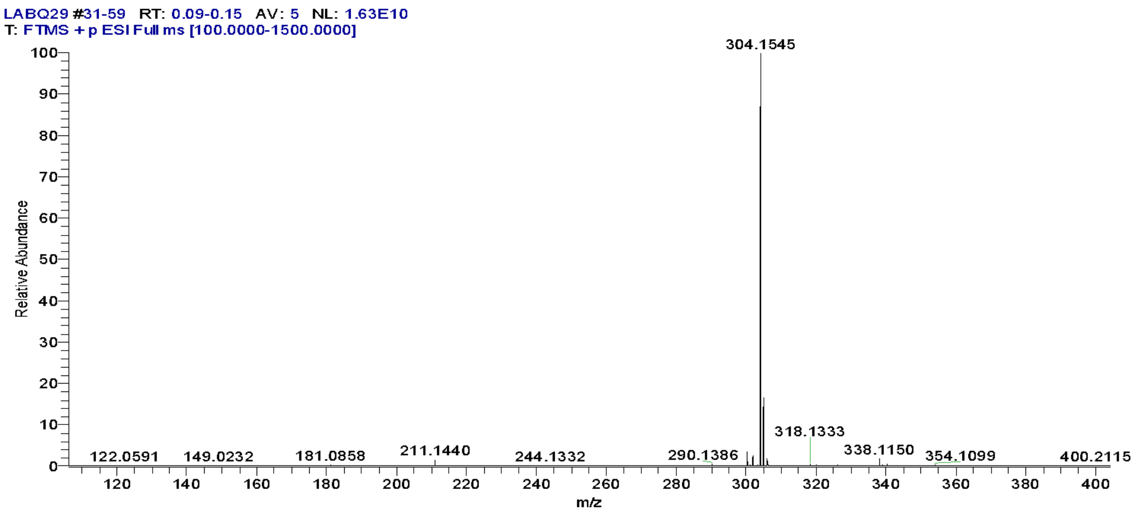

Figure S15. HRMS ESI (+) spectrum of *m/z* 304.1545 $[M+H]^+$ **(compound 3a)**.

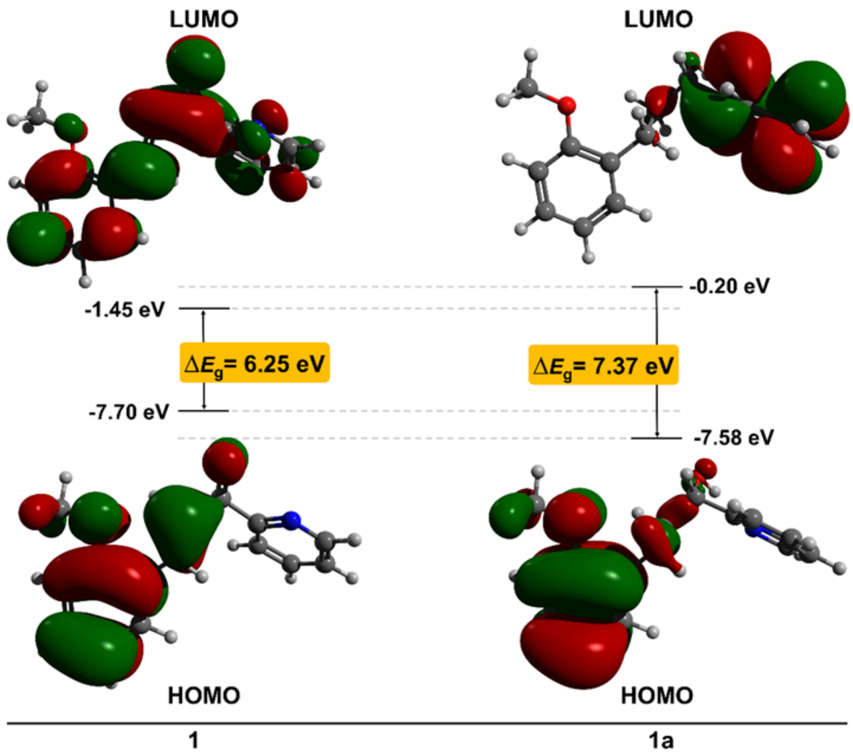

Figure S16. HOMO and LUMO for compounds 1 and 1a.

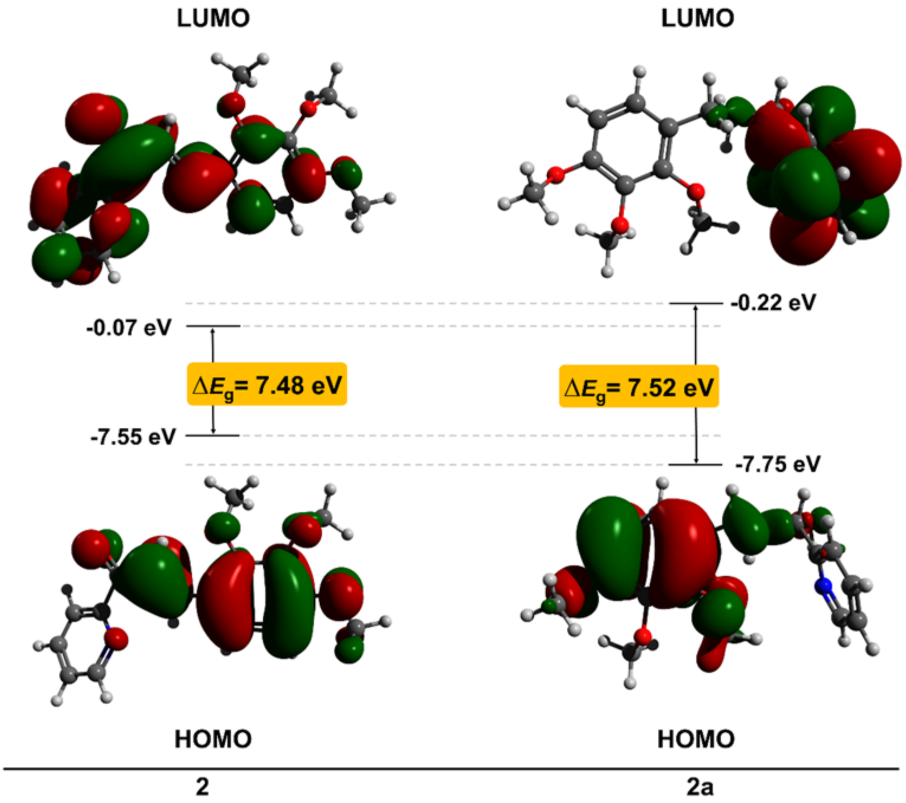

Figure S17. HOMO and LUMO for compounds 2 and 2a.

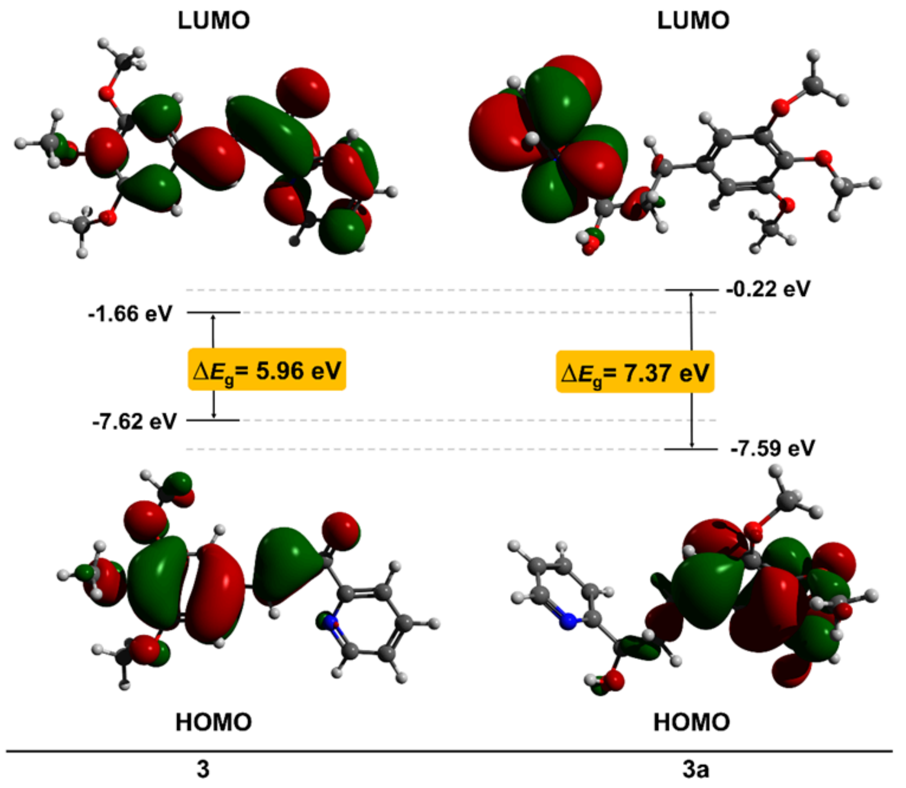

Figure S18. HOMO and LUMO for compounds 3 and 3a.
